# Supplementary material for: Nicotinamide Mononucleotide Administration Prevents Experimental Diabetes-Induced Cognitive Impairment and Loss of Hippocampal Neurons
Source: Int J Mol Sci. 2020 May 26;21(11):3756. doi: 10.3390/ijms21113756 (PMC7313029; doi:10.3390/ijms21113756)
Supplement: Supplementary file 1 [file ijms-21-03756-s001.pdf]

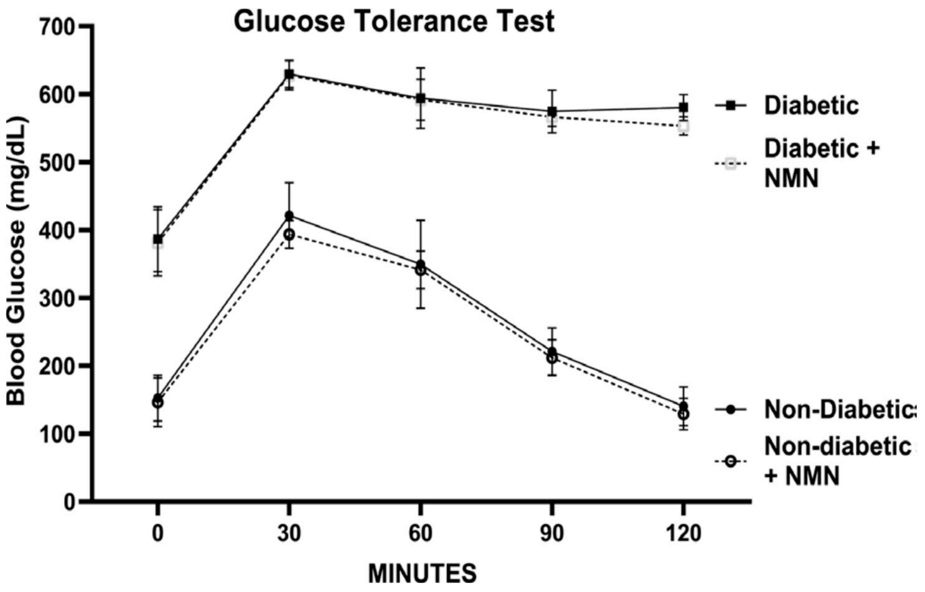

Figure S1. Intraperitoneal glucose tolerance test. The mouse was fasted for 6 hours prior to the glucose tolerance test. The animal had free access to drinking water during the fasting period. A sterile 20 % D-glucose stock solution in water was prepared. Prior to performing a glucose tolerance test, body weight and a baseline glucose level was recorded for each mouse. A small drop of tail blood was placed on the glucometer test strip and the baseline blood glucose value was recorded (in mg/dL). The rat was injected i.p. with a dose of 2 mg glucose/gram body weight. Additional blood samples are obtained at 15, 30, 60, and 120 minutes to measure post-challenge glucose levels. Statistical data for area under the curve for the glucose tolerance test is in Table 1.

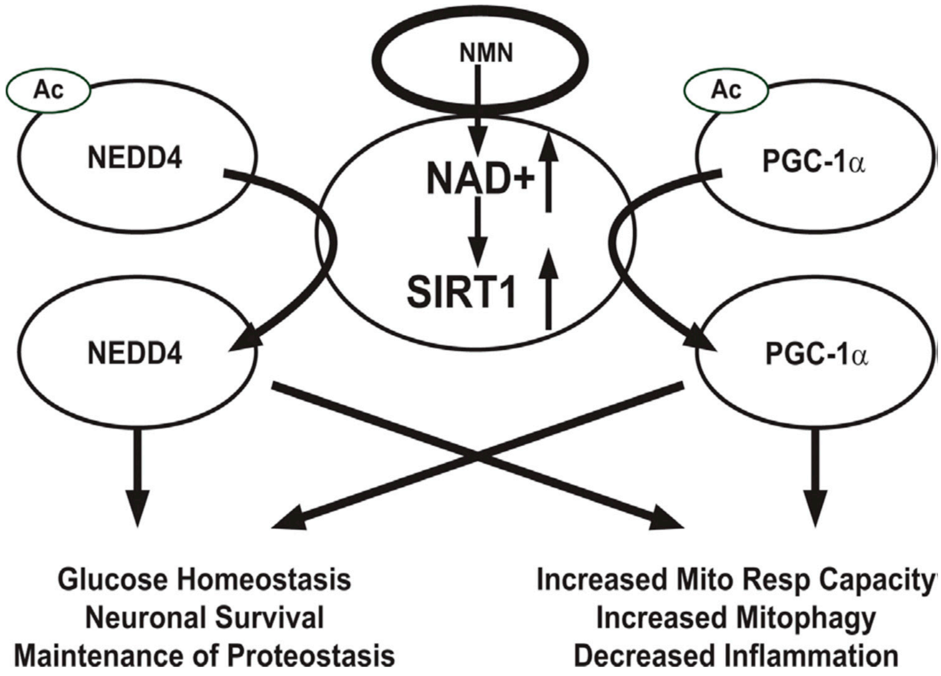

Figure S2. A proposed flow chart on how NMN treatment could prevent diabetes-induced cognitive impairment. NMN administration prevented diabetes-induced decreases in SIRT1 protein level, deacetylates and activates downstream targets PGC-1 alpha and NEDD4. PGC-1 alpha is a transcription factor that promotes mitochondrial biogenesis and oxidative metabolism. NEDD4, a E3 ubiquitin ligase, degrades abnormal mitochondria by activating mitophagy. Mito Resp = Mitochondrial Respiration

Table S1: In gel-extraction and analysis. The region of the immunoblot containing the major acetylated protein band (~100kDa) was identified and placed next to un-transferred remaining gel. The corresponding region of the gel was excised and subjected to in-gel digestion. Briefly, the unstained gel slice was dehydrated with 100% acetonitrile and protein disulfide bonds reduced with 10mM dithiothreitol for 30min at 37°C followed by alkylation of sulfhydryl groups with 20mM 2-chloroacetamide at RT for 30min in the dark using 100mM Tris pH 8.5 buffer. The gel slice was washed with water and dehydrated with 100% acetonitrile prior to adding 12.5ng/ul MS grade Trypsin (Promega) in digestion buffer consisting of 50mM triethylammonium bicarbonate, pH 8, 0.5% sodium deoxycholate. The gel band in enzyme solution was incubated on ice for 45min, excess liquid was subsequently removed and replaced with the above buffer without trypsin and incubated o/n at RT with gentle shaking. Peptides were extracted from the gel and desalted using PolySULFOETHYL A TopTips (PolyLC) to remove interfering substances according to the manufacturer's recommendations. The desalted peptides were analyzed by liquid chromatography tandem mass spectrometry on an LTQ Orbitrap (ThermoFisher). Peptides were separated using a 2hr chromatographic gradient online with a data dependent MS/MS duty cycle of the top 10 most abundant ions. Database search, peptide quantification and identification of acetylated Lysine containing peptides was performed using MaxQuant version

Modifications	Modified sequence	Acetyl (K) Probabilities	Oxidation (M) Probabilities	Acetyl (K)	Acetyl (Protein N-term)	Oxidation (M)	Missed cleavages	Proteins	Gene names	MS/MS m/z	Charge	m/z	Mass
Unmodified	_DDFLGQVDVPLYPLPTENPR_			0	0	0	0	P46935	Nedd4	1143.579	2	1143.076	2284.138
Unmodified	_EGFFELIPQDLIK_			0	0	0	0	P46935	Nedd4	774.9191	2	774.919	1547.824
Unmodified	_ESPENWEIVR_			0	0	0	0	P46935	Nedd4	420.2065	3	420.2069	1257.599
Unmodified	_KDILGASDPYVR_			0	0	0	1	P46935	Nedd4	667.3627	2	667.3592	1332.704
Unmodified	_LAVCGNPATSQPVTSSNHSSR_			0	0	0	0	P46935	Nedd4	724.3533	3	724.015	2169.023
Unmodified	_LTRDDFLGQVDVPLYPLPTENPR_			0	0	0	1	P46935	Nedd4	886.1372	3	885.7974	2654.37
Unmodified	_SYYVDHNSK_			0	0	0	0	P46935	Nedd4	556.7523	2	556.754	1111.493
2 Acetyl (K)	_TIK(ac)K(ac)SLNPK_	TIK(1)K(1)SLNPK		2	0	0	2	P46935	Nedd4	556.8401	2	556.8373	1111.66
Oxidation (M)	_VTLYDPM(ox)SGILTSVQTK_		VTLYDPM(1)SGILTSVQTK	0	0	1	0	P46935	Nedd4	935.4941	2	934.9873	1867.96
Unmodified	_VTLYDPMSGILTSVQTK_			0	0	0	0	P46935	Nedd4	927.5007	2	926.9899	1851.965
Unmodified	_WILENDPTELDLR_			0	0	0	0	P46935	Nedd4	807.4178	2	807.4121	1612.81
Unmodified	_WNEEILFR_			0	0	0	0	P46935	Nedd4	553.7844	2	553.7851	1105.556
